# Changes in Photosynthetic Characteristics between Green-Leaf Poplar Linn. “2025” and Its Bud-Sporting Colored-Leaf Cultivars

**DOI:** 10.3390/ijms25021225

**Published:** 2024-01-19

**Authors:** Tao Wang, Donghuan Xu, Fan Zhang, Tengyue Yan, Yuhang Li, Zhong Wang, Yinfeng Xie, Weibing Zhuang

**Affiliations:** 1 Jiangsu Key Laboratory for the Research and Utilization of Plant Resources, Institute of Botany, Jiangsu Province and Chinese Academy of Sciences (Nanjing Botanical Garden Mem. Sun Yat-Sen), Nanjing 210014, China; johnwt1007@163.com (T.W.); xu15689774892@163.com (D.X.); zhangfan777@njfu.edu.cn (F.Z.); ytydyx2022@163.com (T.Y.); liyuh1998@163.com (Y.L.); wangzhong@cnbg.net (Z.W.); 2 Co-Innovation Center for Sustainable Forestry in Southern China, College of Life Sciences, Nanjing Forestry University, Nanjing 210037, China; xxyyff@njfu.edu.cn

**Keywords:** colored-leaf poplar, photosynthetic light-response curve, chlorophyll fluorescence, chloroplast ultrastructure, gene expression

## Abstract

Colored-leaf poplar is increasingly popular due to its great ornamental values and application prospects. However, the photosynthetic characteristics of these colored-leaf cultivars have not been well understood. In this study, the photosynthetic differences between green-leaf poplar *Populus deltoids* Linn. “2025” (L2025) and colored-leaf cultivars ‘Zhonghong poplar’ (ZHP), ‘Quanhong poplar’ (QHP), and ‘Caihong poplar’ (CHP) were investigated on several levels, including chloroplast ultrastructure observation, photosynthetic physiological characteristics, and expression analysis of key genes. The results showed that the photosynthetic performance of ZHP was basically consistent with that of L2025, while the ranges of light energy absorption and efficiency of light energy utilization decreased to different degrees in CHP and QHP. A relatively low water use efficiency and high dark respiration rate were observed in QHP, suggesting a relatively weak environmental adaptability. The differences in chloroplast structure in different colored-leaf poplars were further observed by transmission electron microscopy. The disorganization of thylakoid in CHP was considered an important reason, resulting in a significant decrease in chlorophyll content compared with other poplar cultivars. Interestingly, CHP exhibited extremely high photosynthetic electron transport activity and photochemical efficiency, which were conductive to maintaining its relatively high photosynthetic performance. The actual quantum yield of PSII photochemistry of ZHP was basically the same as that of QHP, while the relatively high photosynthetic performance indexes in ZHP suggested a more optimized photosynthetic apparatus, which was crucial for the improvement of photosynthetic efficiency. The differential expressions of a series of key genes in different colored-leaf poplars provided a reasonable explanation for anthocyanin accumulation and specific photosynthetic processes.

## 1. Introduction

*Populus deltoids*, a tall tree in the genus *Populus* and family Salicaceae, is one of the most important fast-growing tree species in the northern hemisphere, which can be used for producing timber, pulp, and paper [1]. *Populus deltoids* Linn. “2025” (L2025) is common in the plains and deserts of northern China, with rich resources and a wide distribution for the protection and commercialization of forests [2]. However, its further popularization and application are limited due to its single leaf color. In recent years, many kinds of colored-leaf poplars with splendid ornamental values, such as ‘Zhonghong poplar’ (ZHP) [3], ‘Quanhong poplar’ (QHP) [4], and ‘Caihong poplar’ (CHP) [5], have been bred from the bud sport of L2025. They are very suitable for urban, garden, and road beautification and bring great economic, social, and ecological benefits [5,6]. However, the growth potential of most cultivars was weaker than that of L2025, and the mechanisms underlying these growth differences have not been fully elucidated.

Photosynthesis, crucial for plant growth, is often affected by leaf coloration. In most cases, colored-leaf plants decrease photosynthetic performance to achieve special ecological functions, such as mimic defense and pollination [7], which could be used to explain their weak growth potential. For this reason, their photosynthetic functions could be maintained by changes in external and internal structures in leaves, such as filtering part of incident light through modification of leaf surface morphology, developing double layers of palisade tissue, or keeping the number of functional chloroplasts in palisade cells [8,9]. Moreover, leaf color mutations including yellow, white, and red leaves are usually caused by different contents and distributions of chlorophyll (*Chl*), carotenoids, and anthocyanins within plants [10]. The decrease in *Chl* content and the destruction of photosystem II (PSII) caused by changes in chloroplast structures were considered the main reasons for the decrease in the photosynthetic rate of colored-leaf plants [11]. Certainly, the photosynthetic capacity of some colored-leaf plants showed only minor changes compared with that of their wild-type with green leaves, while some were even superior to the latter. For example, red and green leaves of poinsettia (*Euphorbia pulcherrima*) showed insignificant differences in photosynthetic capacity despite the strong differences in leaf pigment compositions [12]. Zhou et al. [13] reported a yellow mutant of rice (*Oryza sativa*) induced by gamma rays irradiation, whose photosynthetic performance was significantly higher than its normal green-leaf cultivar. For colored-leaf poplar, study on the relationship between leaf coloration and photosynthetic performance could further promote the breeding of poplar cultivars with high quality and multiple purposes.

Anthocyanins play an important role in various biological processes, including serving as major pigmentation factors for diverse leaf color formations [14]. The genes involved in anthocyanin biosynthesis, including early biosynthetic genes (*CHS*, *CHI*, *F3H*, *F3′H*, and *F3′5′H*) and late biosynthetic genes (*DFR*, *ANS*, and *UFGT*), have been identified from model plants [10,15]. There are also many genes carrying polymorphic SNPs that were found to be involved in the anthocyanin biosynthesis pathway, such as the genes encoding for anthocyanidin glucosyltransferase and the flavonoid galactosyltransferase family [5]. Transcription factors (TFs), such as MYB, bHLH, and WD40, also play important roles in the regulation of leaf colors [16]. In our previous report, the bHLH TFs potentially regulated the expression of genes associated with anthocyanin biosynthesis in *P. deltoids* [1]. Moreover, leaf color mutants also induced a series of differentially expressed genes (DEGs), encoding for chloroplast proteins and controlling enzymes for photosynthesis, such as PSII reaction center D1 and D2 proteins (*PsbA* and *PsbD*) [17], oxygen-evolving enhancer protein (*PsbO* and *PsbP*) [18], core-antenna complexes CP43 and CP47 (*PsbC* and *PsbB*) [19], and chlorophyll a/b binding protein (*Lhca* and *Lhcb*) [20]. These genes could be utilized for characterizing photosynthesis-sensitive colored-leaf genotypes, and thus may be crucial for breeding excellent colored-leaf poplar cultivars and elucidating their photosynthetic characteristics.

In poplar breeding, bud sports are critical materials to improve plant morphological appearances. Among them, ZHP originated from the bud sport of L2025, which showed specific reddish-green leaves distinct from the latter. We have previously reported differences in photosynthetic characteristics between ZHP and L2025, which are mainly due to differences in light use efficiency [2]. In recent years, QHP with the latest reddish-purple leaves and CHP with attractive bright-red leaves have been bred from ZHP, respectively. Their characteristics of having no flocculant and being easy to landscape have promoted their extensive cultivation as landscape trees in China. However, their photosynthetic characteristics have not been fully elucidated, especially the changes in photosynthetic apparatus and the resulting differences in photosynthetic performance mediated by leaf coloration. In the present study, chloroplast structures combined with photosynthetic characteristic parameters and chlorophyll fluorescence, as well as expression levels of related genes, were comprehensively investigated to reveal the photosynthetic characteristics and differences between L2025 and the above three colored-leaf cultivars. The results of this study will provide new insights into scientific cultivation management and the application of colored-leaf poplars.

## 2. Results

### 2.1. Leaf Morphology and Chloroplast Ultrastructure

**Figure 1:**
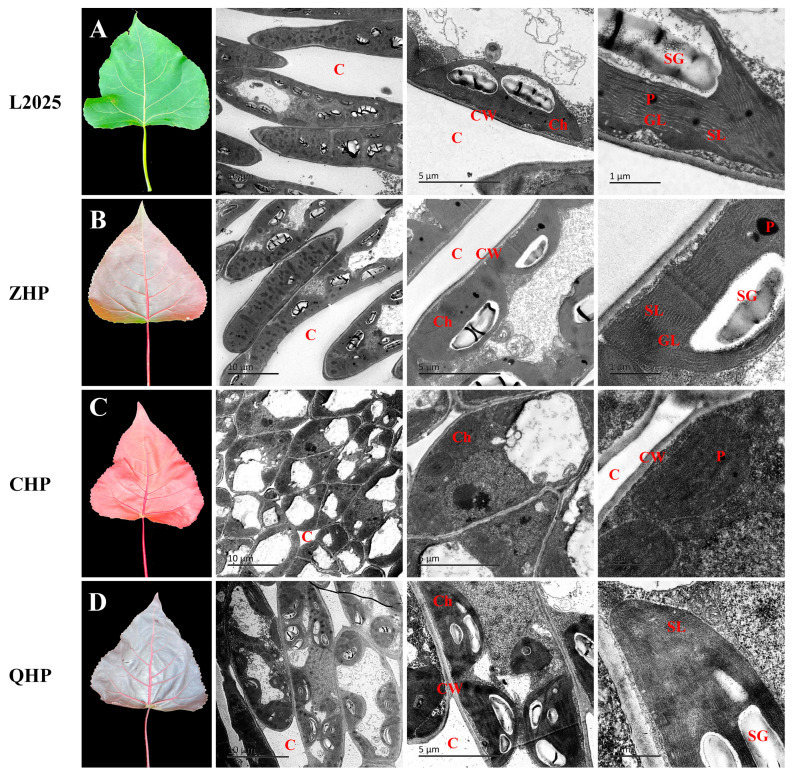
Leaf morphology and chloroplast ultrastructure in different colored poplars. (**A**) L2025; (**B**) ZHP; (**C**) CHP; (**D**) QHP. C, intercellular spaces; Ch, chloroplast; CW, cell wall; GL, grana lamella; SL, stromal lamellae; P, plastoglobuli; SG, starch grain.

As shown in Figure 1, the leaf shape and size in green-leaf poplar L2025 were similar to those in the three colored-leaf cultivars with the same tree ages and branches. However, their leaf color varies greatly. Among them, the leaf color of L2025 is bright green, while ZHP, CHP, and QHP are reddish-green, bright red, and reddish-purple, respectively. The ultrastructural changes in the chloroplast were also observed in different poplar cultivars. Notably, L2025 and ZHP typically exhibit elongated palisade tissue cells, which facilitate their photosynthesis. The palisade tissue cells of QHP were closely arranged, but the cell shape was mostly oval. The shape of mesophyll cells in CHP is irregular, and it is difficult to distinguish the palisade tissues. Moreover, the shape of chloroplasts in L2025 was elongated and oval, and the ultrastructure of chloroplasts was clearly visible, with grana and stroma lamellae orderly embedded in a stromal matrix. Also, a few starch grains and plastoglobuli were observed (Figure 1A). In comparison, the shape of partial chloroplasts in ZHP was slightly irregular while grana stacking remained intact, but thylakoids became swollen. The number of starch grains and plastoglobuli also decreased (Figure 1B). For CHP and QHP, the chloroplasts were decomposed, and the number was sharply reduced, with mostly irregular shapes (from long and oval to elliptical or almost circular). Moreover, the chloroplasts in CHP were decreased in size compared to other cultivars. Notably, the stromal lamellae were clearly visible in QHP, but the grana lamellae were disordered and plastoglobuli had disappeared, as compared to L2025. The membrane structure was well-preserved in CHP, but the lamellae structure was loose and blurry, and the starch grains were almost impossible to observe (Figure 1C,D).

### 2.2. Contents of Photosynthetic Pigment and Anthocyanin in Different Colored-Leaf Poplars

**Figure 2:**
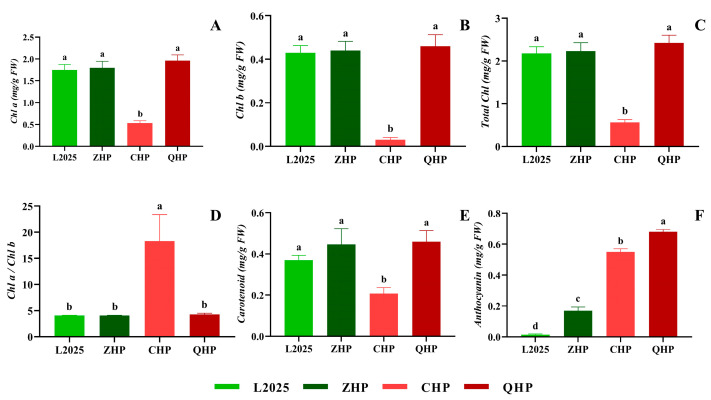
Contents of photosynthetic pigment and anthocyanin in different colored poplars: (**A**) *Chl a*; (**B**) *Chl b*; (**C**) Total *Chl*; (**D**) *Chl a/Chl b*; (**E**) *carotenoid*; (**F**) *anthocyanin*. Data are means ± SE (*n* = 4). Different letters indicate significant differences within different poplar cultivars (*p* < 0.05).

As shown in Figure 2, the *Chl a*, *Chl b*, and carotenoid contents showed no significant differences among L2025, ZHP, and QHP. Correspondingly, there was no significant difference in the total *Chl* content and ratio of *Chl a*/*Chl b* among these three cultivars. However, the *Chl a*/*Chl b* ratio in CHP was significantly higher than those in the mentioned three cultivars, and the other indicators showed the opposite situation (*p* < 0.05). Compared with those in ZHP, the contents of *Chl a*, *Chl b*, carotenoid, and the total *Chl* contents in CHP decreased by 70.29%, 92.97%, 53.59%, and 74.77%, respectively; meanwhile, the ratio of *Chl a*/*Chl b* increased by 3.5 times. Moreover, the anthocyanin content in the four colored poplars was ranked in the order of QHP > CHP > ZHP > L2025, and the values of the different cultivars were significantly different (*p* < 0.05). Among them, the anthocyanin contents of ZHP, CHP, and QHP were increased by 11, 38, and 47 times, respectively, in comparison to L2025.

### 2.3. Measurements of Light Response Curve in Different Colored-Leaf Poplars

**Figure 3:**
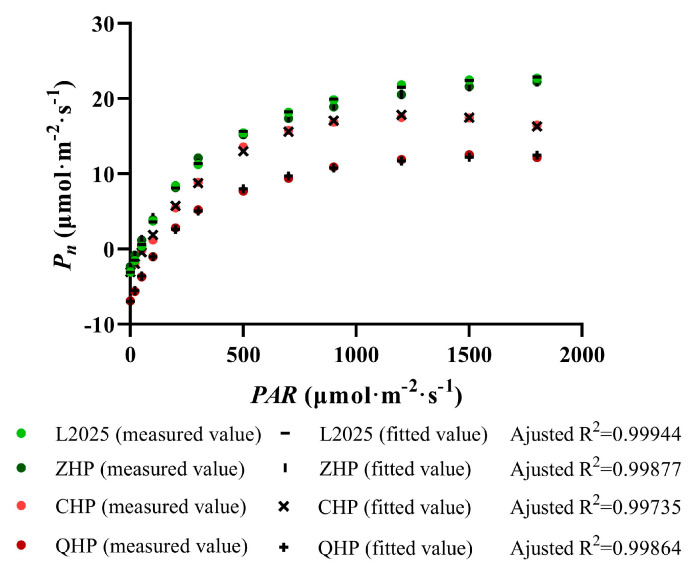
The light response curves and model fit in different colored poplars.

Light intensity plays an important role in the formation of photosynthetic pigments and the development of chloroplasts, and it is considered one of the most important environmental factors affecting photosynthetic performance. In this study, the *Pn* of the four colored poplars showed a similar trend to the continuous increase in *PAR*, which increased rapidly as *PAR* < 300 μmol∙m^−2^∙s^−1^ and increased slowly and finally tended to be gentle as *PAR* > 300 μmol∙m^−2^∙s^−1^ (Figure 3). Among them, the light response curves of L2025 and ZHP were almost coincident; their *Pn* peaked as the *PAR* reached 1500 μmol∙m^−2^∙s^−1^, and were 22.49 and 21.62 μmol∙m^−2^∙s^−1^, respectively. The curve of CHP showed overlaps with L2025 under low-light conditions (*PAR* < 50 μmol∙m^−2^∙s^−1^) and was significantly lower than L2025 thereafter; while the curve of QHP was significantly lower than that of other colored poplars, with the peak value of *Pn* around 12.53 μmol∙m^−2^∙s^−1^ as the *PAR* was up to 1500 μmol∙m^−2^∙s^−1^. Moreover, the measured data were fitted and analyzed with a modified rectangular hyperbolic model, and the determination coefficients (R^2^) of different poplar cultivars were all above 0.997, which indicated that this model fitted very well. This provides a guarantee for the reliability of photosynthetic characteristic parameters fitted by light response curves.

Based on the modified rectangular hyperbolic model, a series of photosynthetic parameters were calculated for evaluation of photosynthetic characteristics in poplars. As shown in Table 1, only the *R_d_* of ZHP decreased by 15.49% compared with that of L2025, and the difference was significant (*p* < 0.05), while the other parameters of ZHP and L2025 showed no significant differences. The *R_d_* of CHP was not significantly different from that of L2025, but the *AQY*, *A_max_*, and *LSP* of CHP decreased by 27.42%, 27.39%, and 49.32%, while the *LCP* increased by 40.45% compared with that of L2025, and the difference was significant (*p* < 0.05). The *R_d_* of QHP was the highest among all poplar cultivars, reaching 6.59 μmol∙m^−2^∙s^−1^; meanwhile, its *LCP* was also the highest, which was more than 2 times higher than that of L2025, and the difference was significant (*p* < 0.05). Moreover, the *LSP* of QHP was significantly higher than that of CHP, but significantly lower than L2025 and ZHP (*p* < 0.05); meanwhile, the *AQY* of QHP was not significantly different from that of CHP.

**Figure 4:**
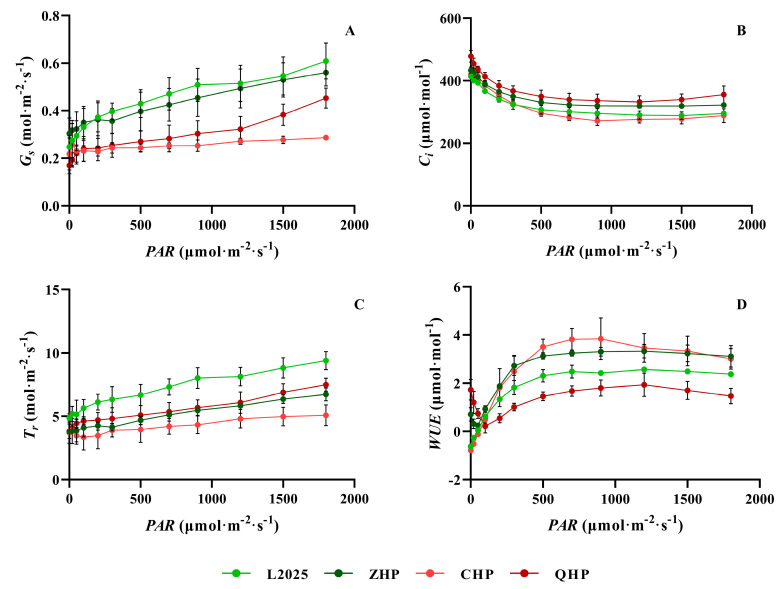
Changes in photosynthetically active radiation (*PAR*) responses from the leaves in different colored poplars: (**A**) stomatal conductance (*Gs*); (**B**) intercellular CO_2_ concentration (*Ci*); (**C**) transpiration rate (*Tr*); (**D**) water use efficiency (*WUE*). Data are means ± SE (*n* = 3).

Moreover, other gas exchange parameters involved in light responses were also measured simultaneously to further evaluate the photosynthetic adaptability of poplar trees. As shown in Figure 4A, the *Gs* of the different poplar cultivars increased gradually with the gradual increase in *PAR*. Among them, the increase in CHP was the smallest, and the value of CHP under different light intensity was also the lowest. The *Gs* of L2025 and ZHP were higher among different poplars, and the difference between them was not significant. The *Gs* of QHP was significantly lower than that of L2025 and ZHP after *PAR* > 300 μmol∙m^−2^∙s^−1^, and significantly higher than that of CHP after PAR > 1000 μmol∙m^−2^∙s^−1^. The trend in *Ci* in different cultivars was opposite to that of *Pn*. With the gradual increase in *PAR*, ZHP and QHP showed higher *Ci* values, and no significant difference was observed within; meanwhile, the *Ci* of L2025 and *Ci* of CHP were significantly lower than the former after *PAR* > 300 μmol∙m^−2^∙s^−1^ (Figure 4B). The trend in *Tr* was similar to that of *Gs*. L2025 and CHP showed the highest and lowest *Tr* values, respectively, under different light intensity with significant difference. The *Tr* of ZHP and QHP showed gradually obvious differences with other poplar cultivars as the increase in *PAR*, while the difference between ZHP and QHP was not significant (Figure 4C). The trend in *WUE* was similar with that of *Pn*. Only ZHP and QHP showed an obvious inflection point for *WUE* under the weak-light condition. Moreover, the *WUEs* of ZHP and CHP were significantly higher than that of L2025 under different light conditions, which was opposite to the situation of QHP (Figure 4D).

### 2.4. Fast Chlorophyll Fluorescence Induction Kinetics in Different Colored-Leaf Poplars

**Figure 5:**
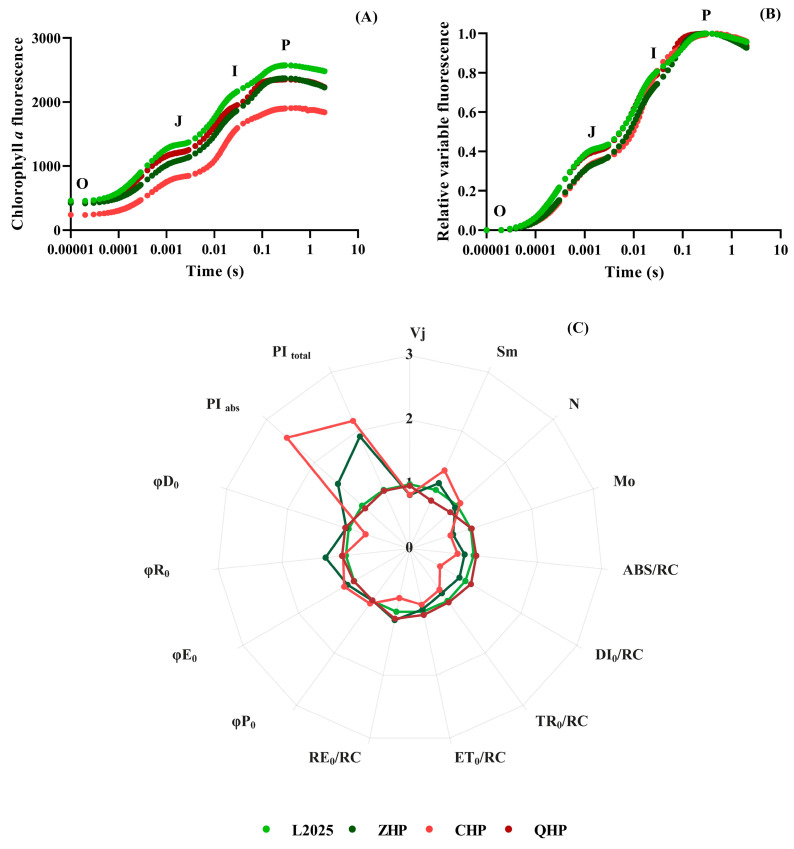
The chlorophyll *a* fluorescence transient kinetic (OJIP) curves (**A**), standardized OJIP curves (**B**), and radar map for chlorophyll *a* fluorescence parameters (**C**).

To further investigate the PSII primary photochemical reaction and changes in the structure and function of photosynthetic apparatus, the chlorophyll *a* fluorescence method was conducted in different colored-leaf poplars. Figure 5A shows the chlorophyll fluorescence induction kinetic curves of different colored-leaf poplars were drawn using the original data, with typical O, J, I, and P phases. However, these curves deviated greatly, especially the fluorescence intensity of L2025 that was significantly higher than CHP during the measurements. Therefore, the fluorescence data were standardized, the fluorescence intensity of the O phase was set to 0, and that of P phase was set to 1, so that all curves have the same starting point and end point, which could be used to accurately compare the differences within different cultivars. Figure 5B shows the standardized OJIP curves of the different cultivars with similar shapes and almost the same time to reach P phase. Notably, the curve difference among poplar cultivars mainly appeared in the J–I phase. The relative variable fluorescence of L2025 and QHP in the J–I phase was significantly higher than that of ZHP and CHP. 

Based on the JIP-test, multiple fluorescence parameters concerning the photosynthetic behavior from the absorption of light by PSII antenna to the reduction in the end electron acceptors driven by PSI could be obtained from the chlorophyll fluorescence kinetic curve. In this study, basic parameters with important physiological significance were selected, and the raw data were normalized to standardize all parameters at the same order of magnitudes. Then, a radar map was drawn with L2025 as a reference to calculate the ratio of other cultivars to L2025 (Figure 5C). Among them, the Vj and Mo of ZHP and CHP were significantly lower than that of L2025, while the Sm of ZHP and CHP increased by 11.47% and 33.13%, respectively, with significant difference. The Sm and N of QHP decreased by 18.25% and 15.31%, and the difference was significant. The results of quantum yield showed that the φE_0_ of ZHP and CHP were significantly higher than that of L2025. The φR_0_ of ZHP increased by 31.77%, while the φD_0_ of CHP decreased by 27.73%, and these changes showed significant differences. The specific energy parameters further reflected the absorption, transformation, and dissipation of light energy in photosynthetic apparatus. The energy fluxes per reaction center of CHP for absorption (ABS/RC), capture (TR_0_/RC), electron transport (ET_0_/RC), dissipation (DI_0_/RC), and reduction of PSⅠ terminal electron acceptors (RE_0_/RC) were significantly lower than that of L2025. The trends in ABS/RC, TR_0_/RC, and DI_0_/RC in ZHP were consistent with those in CHP, but its RE_0_/RC increased by 13.27% with significant difference. The DI_0_/RC and RE_0_/RC of QHP increased by 10.05% and 10.97% compared with L2025, respectively, and the difference was significant. Interestingly, the photosynthetic performance indexes (PI _abs_ and PI _total_) of ZHP were 1.5 times and 1.9 times as large as L2025, respectively; meanwhile, the indexes of CHP were more than twice that of L2025.

### 2.5. Chlorophyll Fluorescence Parameters in Different Colored-Leaf Poplars

**Figure 6:**
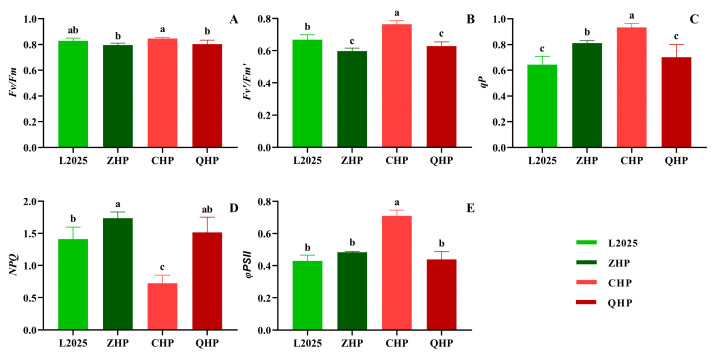
Chlorophyll fluorescence parameters in different colored-leaf poplars: (**A**) *Fv*/*Fm*; (**B**) *Fv*′/*Fm*′; (**C**) *qP*; (**D**) *NPQ*; (**E**) *φPSII*. Data are means ± SE (*n* = 3). Different letters indicate significant differences within different poplar cultivars (*p* < 0.05).

Measurements of steady-state fluorescence can indirectly and harmlessly reflect energy absorption, transfer, and transformation during the photosynthetic process. As shown in Figure 6A, the *Fv/Fm* of different poplars was around 0.8. Among them, the value of three colored-leaf poplars showed no significant difference to that of L2025; meanwhile, the *Fv*/*Fm* of ZHP and QHP was significantly lower than that of CHP (*p* < 0.05). The trend in *Fv*′/*Fm*′ was similar with that in *Fv*/*Fm*. The value in CHP was increased by 14.44% compared with that in L2025, and the difference was significant (*p* < 0.05); meanwhile, the *Fv*′/*Fm*′ of ZHP and QHP were significantly lower than that of L2025 (*p* < 0.05), but with no significant difference between the formers (Figure 6B). The *qP* of CHP was significantly higher than that of other poplars. The value of QHP showed no significant difference from that of L2025 but was significantly lower than that of ZHP (*p* < 0.05) (Figure 6C). The trend in *NPQ* was opposite to the above indicators. Notably, the CHP showed the lowest *NPQ* value among different cultivars, which decreased by 48.67% compared with that of L2025 with significant difference (*p* < 0.05). The *NPQ* of ZHP and QHP were higher than that of L2025 to varying degrees, while the difference between ZHP and L2025 was significant (*p* < 0.05) (Figure 6D). For *φPSII*, the CHP showed the highest value in different cultivars, which increased by 65.29% compared with that of L2025, and the difference was significant (*p* < 0.05); meanwhile, the values in other cultivars showed no significant difference.

### 2.6. Expression Levels of Different Genes in Different Colored-Leaf Poplars

**Figure 7:**
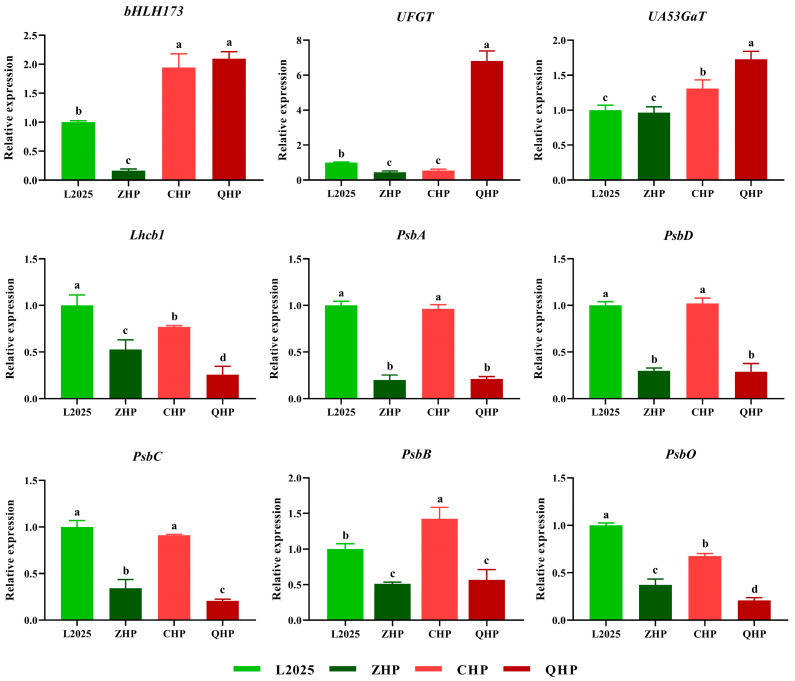
Relative expressions of different genes by qRT–PCR in different colored-leaf poplars. Data are means ± SE (*n* = 3). Different letters indicate significant differences within different poplar cultivars (*p* < 0.05).

As shown in Figure 7, the expressions of *bHLH173* and *UA53GaT* were significantly up-regulated in CHP and QHP, while the former was significantly down-regulated in ZHP compared with that in L2025. The level of *UFGT* was significantly up-regulated in QHP, which was 6.8 times that in L2025. Notably, the levels of photosynthesis-related genes in different poplar cultivars showed a relatively consistent trend. The expressions of these genes were relatively high in L2025 and CHP but were relatively low in ZHP and QHP. Moreover, the expressions of *Lhcb1* and *PsbO* were significantly higher in L2025 than that in CHP, while the situation was reversed for *PsbB,* where the expression in CHP was 1.4 times that of L2025.

## 3. Discussion

The bud-sporting colored-leaf poplar cultivars have excellent characteristics of beautiful tree shapes, heritable leaf color, and no flocculus, which showed promising application prospects in urban landscaping and ecological tourism [6]. However, in their selection and breeding, the photosynthetic performance in functional leaves of the different cultivars showed great differences. The investigation of photosynthetic characteristics in different color-leafed poplars is conducive to their reasonable promotion and utilization in multiple scenes and regions. Among them, the difference in light energy absorption and utilization could be effectively elaborated by photosynthetic light-response curves [21,22]. With the light intensity increased, the Pn of L2025 and ZHP in this study showed no significant difference, but both were higher than that of CHP and QHP; meanwhile, the Pn of CHP was higher than that of QHP. Notably, the photosynthetic performance between L2025 and ZHP was not consistent with our previous report [2], which could be due to the delicate cultivation management and increasing tree ages promoting the adaptability of photosynthetic apparatus to external environment. Moreover, the Gs and Tr of different colored-leaf cultivars decreased to different degrees compared with that of L2025, while the WUE of ZHP and CHP increased significantly, which is believed to be the result of an optimal CO_2_ fixation and water maintenance by stomatal regulation of poplars in response to environmental changes [23]. The QHP showed the lowest WUE and the highest Ci, indicating a weak photosynthetic adaptability to the environment.

The characteristic parameters fitted by the photosynthetic light-response curve could further determine the environmental adaptability of different poplars and evaluate their potential for rapid growth and high yield. In general, functional leaves of plants with relatively high *LSP* and low *LCP* indicate a strong adaptability to light environments [24]. In this study, the *LCP* of CHP and QHP was significantly higher than that of L2025 and ZHP, while the situation of *LSP* was of the opposite, indicating a decreased ability for light energy utilization in these two colored-leaf poplars. Interestingly, the range of available light energy within CHP and QHP was also different, which provided a theoretical basis for their rational cultivation and application. Amax is a direct indicator of the maximum photosynthetic potential of plants, which reflects the growth rate to a certain extent. *AQY* represents the ability of plants to utilize the light energy under weak-light conditions in the morning and evening. Moreover, Rd consumes photosynthetic product to provide energy for plant physiological activities, and cultivars with low *R_d_* values tend to improve productivity [25,26]. In comparison with L2025, the *A_max_* and *AQY* of CHP and QHP decreased significantly, and the Rd of QHP was increased, indicating varying degrees of decreased capacity for photosynthetic product accumulation in the two colored-leaf poplars. These combined results indicate that the light energy-utilization ability and adaptability to adverse environmental factors of different colored-leaf poplar cultivars gradually weakened with the direction of ornamental selection and breeding, which potentially affected their biomass. This was in accordance with the results of growth status for different colored-leaf poplars reported by Gao et al. [27].

The main factors determining plant leaf color are the types and contents of pigments in leaves. For most green-leaf plants, the leaf color is composed of chlorophyll and carotenoids, and leaf color mutations caused by bud mutation are due to chlorophyll deficiency or chloroplast structural mutations that change the ratio of chlorophyll to anthocyanin, leading to changes in leaf color [28]. In this study, the chlorophyll (*Chl a*, *Chl b*, and total *Chl*) and carotenoid contents of QHP showed little difference from those of ZHP and L2025, but the anthocyanin content was significantly higher than the latter two, indicating that the decrease in photosynthetic performance in QHP was not caused by chlorophyll deficiency. Transmission electron microscopy revealed that QHP showed a decreased number of chloroplasts, with swollen chloroplast structure as well as irregular stromal lamellae and grana lamellae, indicating an immature photosynthetic apparatus. The low light energy utilization as well as excess light energy caused by high level of chlorophyll content led to the synthesis of anthocyanins in leaves, which plays a protective role in filtering light energy, eliminating photooxidation stress, and reducing photoinhibition, and is manifested as a low photosynthetic rate [29,30]. Zhu et al. [28] reported that the leaves of colored-leaf poplars could avoid the damage caused by light stress by improving the efficiency of heat dissipation and contributing to non-enzymatic antioxidant capacity; meanwhile, the cultivars with high anthocyanin content could stimulate the leaves to produce a stronger photoprotective effect. This provides a reasonable explanation for the photosynthetic performance of QHP in this study. In comparison, the chlorophyll and carotenoid contents of CHP were significantly lower than those of the other three cultivars, while *Chl a*/*Chl b* and anthocyanins were relatively higher. The observation of ultrastructural chloroplasts further showed the disorganization in thylakoid due to the indistinct stroma and disappearance of grana, and this changed the shape to round or oval. Therefore, an important factor for the overall red appearance of CHP leaves was the significant decrease in chlorophyll content, which could cause the obstruction of chlorophyll synthesis caused by the change in chloroplast structure and, eventually, affect the photosynthetic performance. A previous study has reported that the steps between coprogen III and proto IX are crucial for chlorophyll synthesis in colored-leaf poplars [31]. A similar case was also reported in variegated temple bamboo [32]. Subsequent studies should focus on the identification of blocked sites for chlorophyll synthesis, as these sites may be different in different poplar cultivars, but they all significantly affect chlorophyll content.

The strength of photosynthesis depends not only on the content of photosynthetic pigments but also on the efficiency of photosynthetic electron transport. The JIP-test analysis is based on the theory of energy fluxes in biomembranes, which translates the fluorescence transients into several phenomenological and biophysical expressions for evaluating the stability of photosynthetic apparatus [33,34]. In this study, the pool size of electron carriers per RC (Sm) and the times when Q_A_ reduced to Q_A_^−^ (N) in QHP were both lower than those in L2025, while the situation of specific energy fluxes per RC for dissipation at the level of the antenna chlorophyll (DI_0_/RC) and the reduction at the end of PSI acceptor side (RE_0_/RC) were opposite, which indicated partial inactivation of RC in PSII and relatively weak electron transport from Q_A_ to Q_A_^−^ for QHP, although its PSI performance was enhanced. This phenomenon was related to the reversible state transitions between PSII and PSI, which balanced the distribution of excitation and was required for the functionality of PSI [35]. The trend in indicators in ZHP was similar to our previous reports, which showed a relatively low variable fluorescence intensity at the J-step (Vj), approximated initial slope of the fluorescence transient (Mo), and specific energy fluxes per RC, as well as a relatively high Sm, reduction at the PSI acceptor side (φR_0_), and photosynthetic performance index (PIabs and PItotal), indicating an optimized electron transport system. In comparison, the CHP showed the largest Sm among different cultivars as well as a substantial reduction in DI_0_/RC and the quantum yield of energy dissipation (φD_0_), suggesting lighter quantum were used for electron transport, which also resulted in the highest quantum yield for electron transport (φE_0_), although its specific energy fluxes per RC showed the opposite trend. Moreover, the highest PIabs and PItotal further indicated its excellent electron transport activity and stable photosynthetic apparatus. 

The characteristic parameters based on the OJIP curves only reflect the photosynthetic performance prior to the dark reaction and lack the result under the condition of light adaptation after feedback of the dark reaction. In this study, the photochemical properties of different poplar cultivars were further evaluated under the condition of steady-state fluorescence. Notably, the trend in *Fv/Fm* among different poplar cultivars was basically consistent with that of φP_0_, but there was a slight difference in the significance of parameter values. The latest study reported a light-adapted charge-separated state (PSII_L_) possessed by the PSII photochemical RC. Since the *Fv* is associated with the transition from the closed state PSII_C_ to PSII_L_, the parameter *Fv/Fm* should not be equated with the quantum efficiency of PSII photochemistry [36]. This provides a reasonable explanation for the above results. Moreover, all variables of chlorophyll fluorescence should also be carefully considered when using them to monitor PSII activity and function. Compared with L2025, the *Fv*′*/Fm*′ in both ZHP and QHP were reduced but their *φPSII* were basically the same as that of L2025 when adjusting the openness of PSII RC (*qP*) and energy dissipation (*NPQ*) under suitable environments. This appears to be an effort by different poplars to maintain normal photosynthetic function. Interestingly, the *Fv*′*/Fm*′, *qP*, and *φPSII* of CHP were significantly higher than those of other poplar cultivars, while the NPQ was the opposite, which largely reflected the high level of PSII activity of CHP. It also opens new perspectives for understanding the operation of photosynthetic RCs based on different mutants with different tolerances [37]. Combined with the results of OJIP curves, it is conducive to understand the relatively low chlorophyll content but moderate photosynthetic performance in CHP.

In our previous report, anthocyanin biosynthesis in poplar trees was mainly dependent on the expression of anthocyanin pathway genes regulated by bHLH transcription factors [1]. In the present study, the levels of the representative bHLH family member *bHLH173* as well as anthocyanin pathway genes *UFGT* and *UA53GaT* were all significantly up-regulated in QHP, with the highest anthocyanin content measured in its leaves; furthermore, the levels of *bHLH173* and *UA53GaT* were also up-regulated in CHP with a relatively high anthocyanin content measured within. This indicated that the high expression of anthocyanin pathway genes under the regulation of *bHLH173* was an important approach for the coloration of poplar leaves. However, none of the above genes were up-regulated in ZHP, suggesting other regulatory pathways for the anthocyanin accumulation in ZHP, which reflects the complexity and diversity of leaf-coloration mechanisms. The effect of different leaf-coloration mechanisms on the photosynthetic performance of poplars is an interesting aspect to study in the future. Moreover, PSII, located on the chloroplast thylakoid membrane, is a complex composed of multiple protein subunits. The heterodimers composed of D1-D2 protein constitute the most basic framework of PSII, and various cofactors related to primary charge separation and electron transport are sequentially bound to this framework [38,39]. The levels of *PsbA* and *PsbD* in CHP were consistent with that in L2025; they become an important supplement for the damaged D1 and D2 protein and were crucial for PSII recovery [40,41]. Core antenna complexes CP43 and CP47 are closely linked to D1 and D2 proteins. They are involved in the oxygen evolving process by water-splitting, and can transfer excitation energy captured by LHCII to the reaction center [42]. In this study, the levels of the key genes encoding the above proteins (*PsbB*, *PsbC*, *PsbO*, and *Lhcb1*) in CHP were higher than those of other colored-leaf poplar cultivars. These combined results indicated a high level of LHCII activity and stability of PSII RCs in CHP, which was also consistent with the changes in chlorophyll fluorescence parameters.

## 4. Materials and Methods

### 4.1. Plant Material and Growth Conditions

Three colored-leaf poplar cultivars, ‘ZHP’, ‘CHP’, and ‘QHP’, and a green-leaf poplar cultivar ‘L2025’ were cultivated at the experimental field of Nanjing botanical garden Mem. Sun Yat-Sen, Nanjing, China (32°3′ N, 118°49′ E). This area has a subtropical humid monsoon climate with four distinct seasons. The annual average temperature is approximately 15.4 °C and the average annual precipitation is approximately 1106 mm. The frost-free period is approximately 240 d. In this study, three year-old seedlings per cultivar were planted closely and under the same conditions, such as sunshine and water. The soil used in this field was yellow-brown soil. Eight individuals per cultivar with similar heights and growth conditions were selected, and six mature, fully expanded, and healthy leaves from the third branch (from top to bottom) per plant were marked for subsequent measurements.

### 4.2. Observation of Chloroplast Ultrastructure

For the observation of chloroplast ultrastructure in the leaves of different poplar cultivars, the middle portion of leaves without the midrib was cut into 1–2 mm^2^ pieces and fixed in 4% glutaraldehyde phosphate buffer. Air was removed with a syringe to soak leaves fully in buffer solution. After three rinses of 15 min each with phosphate buffer, they were post-fixed in 2% osmium tetroxide in the same buffer for 2 h. The fixed samples were dehydrated for 30 min in an ascending series of ethanol dilutions of 30%, 50%, 70%, 90%, and 100% (twice). Then, they were impregnated and finally embedded in Epon 812 resin (Sigma, St. Louis, MO, USA). The sections were cut into 50–70 nm with an RMC ultramicrotome and double-stained with uranyl acetate followed by lead citrate. The grids were then examined under a transmission electron microscope JEM-1400 (JEOL, Tokyo, Japan).

### 4.3. Measurement of Chlorophyll and Anthocyanin Contents

The *Chl a*, *Chl b*, total *Chl*, and carotenoid contents in leaves of different poplar cultivars were measured using an 80% acetone extraction method reported by Wang et al. [43]. Samples of 0.1 g were homogenized with 80% acetone, and homogenates were centrifuged at 4 °C for 15 min (6000× *g*). The absorbance of supernatant was measured at 665, 649, and 470 nm by a UV-2102PC/PCS ultraviolet spectrophotometer (UNICO, Shanghai, China). Contents of these pigments were expressed as mg/g fresh weight (FW).

The anthocyanin content in leaves of different poplar cultivars was measured using the method reported by Zhuang et al. [5]. Samples of 0.1 g were immersed into ethanol [containing 1% (*v*/*v*) HCl] at 60 °C for 30 min. After centrifugation, the absorbance of the supernatant was measured at 530, 620, and 650 nm spectrophotometrically. The anthocyanin content was expressed as mg/g fresh weight (FW).

### 4.4. Measurements of Photosynthetic-Light Response Curves

The photosynthetic light-response curves in leaves of different poplar cultivars were measured using an LI-6800 portable photosynthesis system (LI-COR, Lincoln, NE, USA) on the sunny days from 08:30 to 11:30 h. The values of photosynthetically active radiation (PAR) per measurement were successively set to 1800; 1500; 1200; 900; 600; 300; 150; 100; 50; 0 μmol∙m^−2^∙s^−1^. The leaf temperature was set at 25 °C and the relative humidity was set at 50%. A CO_2_ injection system was used to provide a constant CO_2_ concentration of 400 μmol∙mol^−1^ in the sample chamber. The measured parameters included net photosynthetic rate (*Pn*), stomatal conductance (*Gs*), intercellular CO_2_ concentration (*Ci*), transpiration rate (*Tr*), and water use efficiency (*WUE*), which was calculated as *Pn*/*Tr*. Three marked leaves from different individuals per cultivar were used for the measurements.

The measured curves were fitted by a modified rectangular hyperbolic model reported by Ye et al. [21]. Parameter estimation and calculation were conducted by an online tool (http://photosynthetic.sinaapp.com/calc.html accessed on 20 September 2023). Among them, *LCP* represents the light compensation point; *LSP* represents the light saturation point; *A_max_* is the maximum net photosynthetic rate; *AQE* is the apparent quantum efficiency; and *R_d_* is the dark respiration rate.

### 4.5. Measurements of Chlorophyll Fluorescence

The *Chl a* fluorescence transient in leaves of different poplar cultivars was measured using a Handy PEA (Hansatech, Kings Lynn, UK). Firstly, all the measured leaves were dark-adapted for 30 min by clips fixing on the central position of the marked leaves. Then, four marked leaves from different individuals per cultivar were used for the measurements with the PEA probe, and the data were analyzed by the JIP-test [33], which provided a large amount of information about the donor side, the acceptor side, and the reaction center (RC) of PSII. The basic fluorescence parameters were listed in Table 2:

The steady-state fluorescence in leaves of different poplar cultivars was obtained using a CF-Imaging system (CF Imager, Technologica, Essex, UK) following the manufacturer’s instructions. Four detached leaves from different individuals per cultivar were dark-adapted for 30 min in a measuring chamber. After evaluating the dark-adapted minimum fluorescence (*Fo*) and dark-adapted maximum fluorescence (*Fm*), the actinic light of 600 µmol (photon) m^−2^ s^−1^ was switched on with saturating pulses of 1800 µmol (photon) m^−2^ s^−1^ repeated every 25 s for evaluation of steady-state fluorescence (*Fs*) and light-adapted maximum fluorescence (*Fm*′). The light-adapted minimum fluorescence (*Fo*′) was recorded after 3 s of far-red light illumination as the actinic light was switched off. The fluorescence parameters including the maximum quantum efficiency of PSII photochemistry (*Fv/Fm*), effective quantum yield of PSII photochemistry (*Fv*′*/Fm*′), non-photochemical quenching (*NPQ*), photochemical quenching coefficient (*qP*), and actual quantum yield of PSII photochemistry (*φPSII*) were obtained by the internal software Fluor Imager (version 2.2). To facilitate comparison of the above measurements with those in the other literature, the conventional chlorophyll fluorescence terms were used in this study, and the latest and most accurate interpretation of the measured parameters was referred to in the discussions.

### 4.6. Measurements of qRT-PCR

The samples of different poplar cultivars were first prepared for RNA isolation. Total RNA was isolated from 0.1 g of crushed leaves with a plant RNA kit (Huayueyang, Beijing, China) according to the manufacturer’s protocol. Then, the first strand of cDNA was synthesized from 1 μg of total RNA using a PrimeScript RT reagent Kit (Takara, Dalian, China). The qPCR was performed in an Applied Biosystems 7500 Real-Time PCR System (Applied Biosystems, Waltham, MA, USA) with SYBR Green II PCR Master Mix (Takara, Dalian, China). The reaction was carried out in a final volume of 20 μL (containing 4 μL of cDNA) with the following conditions: initial denaturation at 95 °C for 30 s, 40 cycles of denaturation at 95 °C for 5 s, and annealing and extension at 60 °C for 34 s. A melting curve was obtained at 95 °C for 1 s and at 60 °C for 1 min followed by continuous heating. Finally, the qRT-PCR results were analyzed with the REST 2009 software (version 2.0.13). In this study, the primers for the corresponding genes were designed on primer 5, and actin2 was used as an internal control (Table 3).

### 4.7. Statistical Analysis

The results were expressed as mean ± SE of at least three biological replicates. Statistical analysis was conducted using SPSS software version 19.0 and Microsoft Excel 2019. Multiple comparison analyses were performed using one-way analysis of variance (ANOVA) with Duncan’s test (*p* < 0.05). Data normality and variance homogeneity were tested with Shapiro and Levene’s tests in SPSS 19.0 prior to the statistical analysis. The graphs were produced using ChiPlot (https://www.chiplot.online/ accessed on 20 September 2003) and GraphPad Prism 8.0.

## 5. Conclusions

The assessment of these results allows us to conclude that the photosynthetic performance of different colored-leaf poplars showed obvious differences during the photosynthetic light response process. Among them, the levels of most photosynthetic parameters in ZHP were basically consistent with that in L2025; meanwhile, the light energy absorption range and utilization efficiency in CHP and QHP decreased to different degrees, and the levels between them were also different. According to the results of leaf pigment content, the anthocyanin content of CHP and QHP was sharply increased compared with that of L2025, and the chlorophyll content of CHP was significantly lower than that of other poplar cultivars. Combined with the observation of chloroplast ultrastructure, it is speculated that light filtering by anthocyanin accumulation and the obstruction of chlorophyll synthesis induced by chloroplast structure changes were the main factors for the decrease in photosynthetic performance of QHP and CHP, respectively. Notably, CHP exhibited extremely high photosynthetic electron transport activity and photochemical efficiency; ZHP also exhibited relatively high PSII activity. This was thought to be the key to improving photosynthetic performance. Moreover, the *φPSII* of ZHP, QHP, and L2025 were basically the same when adjusting the openness of PSII RC and energy dissipation, which was conductive to their normal photosynthetic functions. In the present study, the photosynthetic characteristics and differences between L2025 and its bud-sporting colored-leaf cultivars were investigated, and contribute to a comprehensive understanding of anthocyanin-mediated changes in the photosynthetic performance of colored-leaf poplars. Further research should be deeply carried out on the photosynthetic mechanism at the molecular level, as this will lay a foundation for directive breeding, promotion, and utilization of excellent poplar cultivars.

## Figures and Tables

**Table 1:** Photosynthetic characteristic parameters in different colored poplars.

	*AQY*	*A_max_* (μmol∙m^−2^∙s^−1^)	*R_d_* (μmol∙m^−2^∙s^−1^)	*LSP* (μmol∙m^−2^∙s^−1^)	*LCP* (μmol∙m^−2^∙s^−1^)
L2025	0.062 ± 0.004 a	22.621 ± 3.120 a	3.124 ± 0.046 b	2408.087 ± 154.371 a	40.915 ± 0.436 c
ZHP	0.060 ± 0.002 a	22.535 ± 1.816 a	2.640 ± 0.234 c	2739.033 ± 318.509 a	34.501 ± 3.677 c
CHP	0.045 ± 0.006 b	16.425 ± 1.275 b	3.149 ± 0.274 b	1220.310 ± 171.868 c	57.464 ± 3.004 b
QHP	0.048 ± 0.008 b	11.216 ± 1.210 c	6.594 ± 0.392 a	1858.683 ± 265.537 b	126.374 ± 5.138 a

Data are means ± SE (*n* = 3). Different letters indicate significant differences within the same column (*p* < 0.05).

**Table 2:** OJIP-test parameters in the study.

Fluorescence Parameter	Description
Derived parameters	
Vj	relative variable fluorescence at the J-step
Sm	normalized total complementary area above the OJIP transient or total electron carriers per RC
N	the times Q_A_ was reduced to Q_A_^−^ in the time span from t_0_ to t_Fmax_
Mo	approximated initial slope of the *Chl* fluorescence transient V = f(t)
Specifc fuxes or activities per RC	
ABS/RC	average absorbed photon flux per PSII RC
TR_0_/RC	specific energy fluxes per RC for trapping at t = 0
ET_0_/RC	specific energy fluxes per RC for electron transport at t = 0
DI_0_/RC	specific energy fluxes per RC for dissipation at t = 0
RE_0_/RC	specific electron fluxes per RC for reduction of PSI acceptors at t = 0
Yields or fux ratios	
φP_0_	maximum quantum yield of primary PSII photochemistry
φE_0_	quantum yield for electron transport from Q_A_^−^ to plastoquinone
φD_0_	quantum yield (t = 0) of energy dissipation
φR_0_	quantum yield for reduction in the end electron acceptors at the PSI acceptor side
Performance index	
PI_abs_	performance index for energy conservation from photons absorbed by PSII antenna to the reduction of Q_B_
PI_total_	performance index for energy conservation from photons absorbed by PSII antenna to the reduction of PSI acceptors

**Table 3:** Specific primers used in relative qRT-PCR.

Gene Name	Accession	Description	Forward Primer (5′ to 3′)	Reverse Primer (5′ to 3′)
*PsbA*	Potri.013G143200	photosystem II reaction center protein A (D1)	GGGTCGCTTCTGTAATTGG	AGTTGCGGTCAATAAAGTAGG
*PsbD*	Potri.008G208600	photosystem II reaction center protein D (D2)	TGCAATCGCATTCTCTGG	AACTAGGCGCAAAGAACC
*PsbC*	Potri.010G032700	photosystem II reaction center protein C (CP43)	GGAAGTCATAGACACCTTTCC	TCGGGTCCTAGAAGTGC
*PsbB*	Potri.011G113900	photosystem II reaction center protein B (CP47)	GATAAAGAAGGGCGTGAGC	ATTCCGTCTCCGTCTACC
*PsbO*	Potri.005G130400	photosystem II oxygen-evolving enhancer protein 1	TGAAGGAGTTCCGAAGAGG	TCAATGGTTGGGCATTGG
*Lhcb1*	Potri.005G239200	light-harvesting complex II chlorophyll a/b binding protein 1 (lhcb1)	ATCACTGACCCGATCTACC	CAGTCTACCATTCTTGAGTTCC
*UFGT*	Potri.009G133300	Flavonoid 3-O-galactosyl transferase family protein	TGGCGTATATCAGCTTTGG	CCTAAGAGACCAAAGGAATGG
*UA53GaT*	Potri.015G027700	Anthocyanidin 5,3-O-glucosyltransferase	AAACGGCTATTGGGTTGG	TCATTTGGAGTGCTTGACC
*bHLH173*		bHLH transcription factor	CCTCGAATGTGAGGAAACC	AAACTGAACTTCCTTCCTAGC
*Actin2*		Actin	GCCATCTCTCATCGGAATGGAA	AGGGCAGTGATTTCCTTGCTCA

## Data Availability

The data presented in this study are available on request from the corresponding author.

## References

[1] Wang X.-J., Peng X.-Q., Shu X.-C., Li Y.-H., Wang Z., Zhuang W.-B. (2022). Genome-wide identification and characterization of PdbHLH transcription factors related to anthocyanin biosynthesis in colored-leaf poplar (*Populus deltoids*). BMC Genom..

[2] Wang T., Li L., Cheng G., Shu X., Wang N., Zhang F., Zhuang W., Wang Z. (2021). Physiological and molecular analysis reveals the differences of photosynthesis between colored and green leaf poplars. Int. J. Mol. Sci..

[3] Chen M., Chang C., Li H., Huang L., Zhou Z., Zhu J., Liu D. (2023). Metabolome analysis reveals flavonoid changes during the leaf color transition in Populus× euramericana ‘Zhonghuahongye’. Front. Plant Sci..

[4] Zhang F., Wan X., Zheng Y., Sun L., Chen Q., Guo Y., Zhu X., Liu M. (2014). Physiological and related anthocyanin biosynthesis genes responses induced by cadmium stress in a new colored-leaf plant “Quanhong Poplar”. Agrofor. Syst..

[5] Zhuang W., Wang H., Liu T., Wang T., Zhang F., Shu X., Zhai H., Wang Z. (2019). Integrated physiological and genomic analysis reveals structural variations and expression patterns of candidate genes for colored-and green-leaf poplar. Sci. Rep..

[6] Zhang F., Zhao J., Wan X., Luo X., Li W., Sun L., Chen Q. (2016). From green to red: Large-scale transcriptome comparison of a bud sport in poplar (*Populus deltoides*). Acta Physiol. Plant..

[7] Zhang K., Wang X., Cui J., Ogweno J., Shi K., Zhou Y., Yu J. (2011). Characteristics of gas exchange and chlorophyll fluorescence in red and green leaves of Begonia semperflorens. Biol. Plant..

[8] Karabourniotis G., Bornman J., Liakoura V. (1999). Different leaf surface characteristics of three grape cultivars affect leaf optical properties as measured with fibre optics: Possible implication in stress tolerance. Funct. Plant Biol..

[9] Liakopoulos G., Nikolopoulos D., Klouvatou A., Vekkos K.-A., Manetas Y., Karabourniotis G. (2006). The photoprotective role of epidermal anthocyanins and surface pubescence in young leaves of grapevine (*Vitis vinifera*). Ann. Bot..

[10] Tanaka Y., Sasaki N., Ohmiya A. (2008). Biosynthesis of plant pigments: Anthocyanins, betalains and carotenoids. Plant J..

[11] Hanke G., Mulo P. (2013). Plant type ferredoxins and ferredoxin-dependent metabolism. Plant Cell Environ..

[12] Pomar F., Ros Barceló A. (2007). Are red leaves photosynthetically active?. Biol. Plant..

[13] Zhou X.-S., Shen S.-Q., Wu D.-X., Sun J.-W., Shu Q.-Y. (2006). Introduction of a xantha mutation for testing and increasing varietal purity in hybrid rice. Field Crops Res..

[14] Wen W., Alseekh S., Fernie A.R. (2020). Conservation and diversification of flavonoid metabolism in the plant kingdom. Curr. Opin. Plant Biol..

[15] Routaboul J.-M., Kerhoas L., Debeaujon I., Pourcel L., Caboche M., Einhorn J., Lepiniec L. (2006). Flavonoid diversity and biosynthesis in seed of Arabidopsis thaliana. Planta.

[16] Outchkourov N.S., Karlova R., Hölscher M., Schrama X., Blilou I., Jongedijk E., Simon C.D., van Dijk A.D., Bosch D., Hall R.D. (2018). Transcription factor-mediated control of anthocyanin biosynthesis in vegetative tissues. Plant Physiol..

[17] Kós P.B., Deák Z., Cheregi O., Vass I. (2008). Differential regulation of psbA and psbD gene expression, and the role of the different D1 protein copies in the cyanobacterium Thermosynechococcus elongatus BP-1. Biochim. Biophys. Acta (BBA)-Bioenerg..

[18] Jain A., Cao A., Karthikeyan A., Baldwin J., Raghothama K. (2005). Phosphate deficiency suppresses expression of light-regulated psbO and psbP genes encoding extrinsic proteins of oxygen-evolving complex of PSII. Curr. Sci..

[19] Murray J.W., Duncan J., Barber J. (2006). CP43-like chlorophyll binding proteins: Structural and evolutionary implications. Trends Plant Sci..

[20] Pan X., Cao P., Su X., Liu Z., Li M. (2020). Structural analysis and comparison of light-harvesting complexes I and II. Biochim. Biophys. Acta (BBA)-Bioenerg..

[21] Ye Z.P., Suggett D.J., Robakowski P., Kang H.J. (2013). A mechanistic model for the photosynthesis–light response based on the photosynthetic electron transport of photosystem II in C3 and C4 species. New Phytol..

[22] Li Y., Liu X., Hao K., Yang Q., Yang X., Zhang W., Cong Y. (2019). Light-response curve of photosynthesis and model fitting in leaves of Mangifera indica under different soil water conditions. Photosynthetica.

[23] Vialet-Chabrand S.R., Matthews J.S., McAusland L., Blatt M.R., Griffiths H., Lawson T. (2017). Temporal dynamics of stomatal behavior: Modeling and implications for photosynthesis and water use. Plant Physiol..

[24] Liu M., Qi H., Zhang Z., Song Z., Kou T., Zhang W., Yu J. (2012). Response of photosynthesis and chlorophyll fluorescence to drought stress in two maize cultivars. Afr. J. Agric. Res..

[25] Liu X., Fan Y., Long J., Wei R., Kjelgren R., Gong C., Zhao J. (2013). Effects of soil water and nitrogen availability on photosynthesis and water use efficiency of Robinia pseudoacacia seedlings. J. Environ. Sci..

[26] Zhu J., Wang K., Sun Y., Yan Q. (2014). Response of Pinus koraiensis seedling growth to different light conditions based on the assessment of photosynthesis in current and one-year-old needles. J. For. Res..

[27] Gao M., Zhao Y., Zong Y., Wang W. (2020). Photosynthetic Traits and Ecological Adaptability of Poplar 2025 and Its 3 Bud Sporting Color-leafed Cultivars. J. Northwest For. Univ..

[28] Zhu X., Yang J., Wen D., Han X., Ru G. (2020). Photoprotective effects of anthocyanins in leaves of color-leaved poplar. Southwest China J. Agric. Sci..

[29] Zeng X.Q., Chow W.S., Su L.J., Peng X.X., Peng C.L. (2010). Protective effect of supplemental anthocyanins on Arabidopsis leaves under high light. Physiol. Plant..

[30] Gould K.S., Jay-Allemand C., Logan B.A., Baissac Y., Bidel L.P. (2018). When are foliar anthocyanins useful to plants? Re-evaluation of the photoprotection hypothesis using Arabidopsis thaliana mutants that differ in anthocyanin accumulation. Environ. Exp. Bot..

[31] Zhang S., Wu X., Cui J., Zhang F., Wan X., Liu Q., Zhong Y., Lin T. (2019). Physiological and transcriptomic analysis of yellow leaf coloration in Populus deltoides Marsh. PLoS ONE.

[32] Chen L., Lai J., He T., Rong J., Tarin M.W.K., Zheng Y. (2018). Differences in photosynthesis of variegated temple bamboo leaves with various levels of variegation are related to chlorophyll biosynthesis and chloroplast development. J. Am. Soc. Hortic. Sci..

[33] Strasser R.J., Tsimilli-Michael M., Srivastava A. (2004). Analysis of the chlorophyll a fluorescence transient. Chlorophyll a Fluorescence: A Signature of Photosynthesis.

[34] Alexandrina Stirbet G. (2011). On the relation between the Kautsky effect (chlorophyll a fluorescence induction) and Photosystem II: Basics and applications of the OJIP fluorescence transient. J. Photochem. Photobiol. B Biol..

[35] Wang T., Luo S., Ma Y., Li L., Xie Y., Zhang W. (2020). Chlorophyll a Fluorescence Transient and 2-Dimensional Electrophoresis Analyses Reveal Response Characteristics of Photosynthesis to Heat Stress in Malus.‘Prairifire’. Plants.

[36] Sipka G., Magyar M., Mezzetti A., Akhtar P., Zhu Q., Xiao Y., Han G., Santabarbara S., Shen J.-R., Lambrev P.H. (2021). Light-adapted charge-separated state of photosystem II: Structural and functional dynamics of the closed reaction center. Plant Cell.

[37] Garab G., Magyar M., Sipka G., Lambrev P.H. (2023). New foundations for the physical mechanism of variable chlorophyll a fluorescence. Quantum efficiency versus the light-adapted state of photosystem II. J. Exp. Bot..

[38] Gao J., Wang H., Yuan Q., Feng Y. (2018). Structure and function of the photosystem supercomplexes. Front. Plant Sci..

[39] Nixon P.J., Michoux F., Yu J., Boehm M., Komenda J. (2010). Recent advances in understanding the assembly and repair of photosystem II. Ann. Bot..

[40] Pospíšil P. (2012). Molecular mechanisms of production and scavenging of reactive oxygen species by photosystem II. Biochim. Biophys. Acta (BBA)-Bioenerg..

[41] Jansen M.A., Gaba V., Greenberg B.M., Mattoo A.K., Edelman M. (1996). Low threshold levels of ultraviolet-B in a background of photosynthetically active radiation trigger rapid degradation of the D2 protein of photosystem-II. Plant J..

[42] Bi A., Fan J., Hu Z., Wang G., Amombo E., Fu J., Hu T. (2016). Differential acclimation of enzymatic antioxidant metabolism and photosystem II photochemistry in tall fescue under drought and heat and the combined stresses. Front. Plant Sci..

[43] Wang T., Li L., Qin Y., Lu B., Xu D., Zhuang W., Shu X., Zhang F., Wang N., Wang Z. (2023). Effects of seasonal changes on chlorophyll fluorescence and physiological characteristics in the two Taxus species. Plants.

